# Variation in Macro and Trace Elements in Progression of Type 2 Diabetes

**DOI:** 10.1155/2014/461591

**Published:** 2014-08-05

**Authors:** Khalid Siddiqui, Nahla Bawazeer, Salini Scaria Joy

**Affiliations:** ^1^Strategic Center for Diabetes Research, King Saud University, P.O. Box 245, Riyadh 11411, Saudi Arabia; ^2^Nutrition Department, University Diabetes Center, King Saud University, P.O. Box 245, Riyadh 11411, Saudi Arabia

## Abstract

Macro elements are the minerals of which the body needs more amounts and are more important than any other elements. Trace elements constitute a minute part of the living tissues and have various metabolic characteristics and functions. Trace elements participate in tissue and cellular and subcellular functions; these include immune regulation by humoral and cellular mechanisms, nerve conduction, muscle contractions, membrane potential regulations, and mitochondrial activity and enzyme reactions. The status of micronutrients such as iron and vanadium is higher in type 2 diabetes. The calcium, magnesium, sodium, chromium, cobalt, iodine, iron, selenium, manganese, and zinc seem to be low in type 2 diabetes while elements such as potassium and copper have no effect. In this review, we emphasized the status of macro and trace elements in type 2 diabetes and its advantages or disadvantages; this helps to understand the mechanism, progression, and prevention of type 2 diabetes due to the lack and deficiency of different macro and trace elements.

## 1. Introduction

Diabetes is a chronic disease that occurs either when the pancreas does not produce enough insulin or when the body cannot effectively use the insulin it produces [1]. The prevalence of diabetes in the age groups between 20 to 70 years worldwide was estimated to be 8.3% in 2013 and 10.1% in 2035. The total number of adult with diabetes is projected to rise from 382 million in 2013 to 592 million in 2035. In 2013, an estimated 5.1 million people died from consequences of hyperglycemia. More than 80% of diabetes deaths occur in low- and middle-income countries. According to the International Diabetes Federation (IDF), the cost for the treatment of diabetes globally in 2010 was about $ 376 billion (11.6 percent of total health spending). The IDF predicts that these costs will increase by 2030 to $ 490 billion and it puts considerable strain on health systems. Type 2 diabetes can be prevented or delayed through healthy diet, regular physical activity, maintaining a normal body weight, and avoiding smoking [2].

The aethiological types of disorders of glycemia includes Type 1 diabetes (T1D), Type 2 diabetes (T2D), and gestational diabetes mellitus (GDM). T1D indicates the process of *β*-cell destruction that may ultimately lead to diabetes mellitus in which “insulin is required for survival” to prevent the development of ketoacidosis, coma, and death. T2D is the most common form of diabetes and individuals are characterized by disorders of insulin action and secretion, either of which may be the predominant feature. Gestational diabetes is carbohydrate intolerance resulting in hyperglycemia of variable severity with onset or first recognition during pregnancy [3].

**Figure 1:**
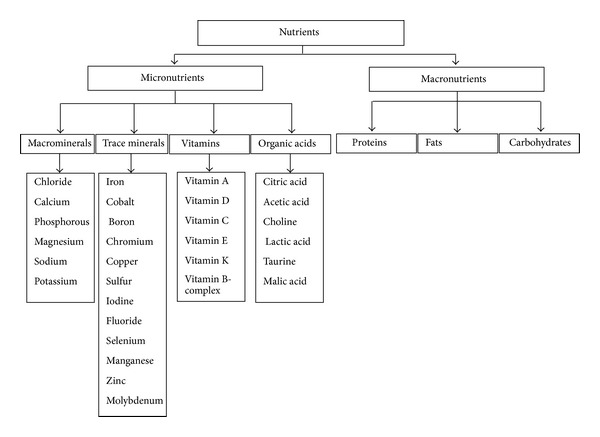
Classification of different nutrients need in metabolism.

Micronutrients are essential nutrients that are required by the body in trace amounts or tiny quantities on a day-to-day basis in order to function properly. This includes four major classes: macro elements, trace elements, vitamins, and organic acids. Macro elements include chloride, calcium, phosphorous, magnesium, sodium, potassium, and iron. The trace elements include cobalt, boron, chromium, copper, sulfur, iodine, fluoride, selenium, manganese, zinc, and molybdenum (Figure 1).

Macro elements have multiple roles within the body. They work together with vitamins and initiate hormone production as well as speeding up the metabolic processes. Trace elements participate in tissue and cellular and subcellular functions; these include immune regulation by humoral and cellular mechanisms, nerve conductions, muscle contractions, membrane potential regulations, mitochondrial activity, and enzyme reactions. Trace elements interact with vitamins and macro elements to enhance their effects on the body. They are accepted as essential for human health and have diverse metabolic characteristics and functions [4].

Direct associations of macro and trace elements with diabetes mellitus (DM) have been observed in many research studies [5]. Insulin action on reducing blood glucose was reported to be potentiated by some trace elements as chromium, magnesium, vanadium zinc, manganese, molybdenum, and selenium [6]. The proposed mechanism of trace elements enhancing insulin action includes activation of insulin receptor sites, serving as cofactors or components for enzyme systems involved in glucose metabolism [7], increasing insulin sensitivity, and acting as antioxidants preventing tissue peroxidation [8]. It is also reported that the metabolism of several trace and macro elements alters T2D and these elements might have specific roles in the pathogenesis and progress of this disease. Here we emphasize the status of macro elements and trace elements those have been reported to be either involved in glucose homeostasis or their levels modulated in T2D. This paper also analyzes the status of evidence for the selected micronutrients in T2D (Table 1), its advantages, disadvantages, mechanism of action, progression or prevention of disease and function in type 2 diabetes (Table 2).

## 2. Macro Elements

Macro elements are the natural elements of which the body needs more amount and are more important than any other minerals. Macrominerals includes sodium (Na), potassium (K), calcium (Ca), and magnesium (Mg) which are cations; and two chlorine (Cl) and phosphorus (P) which are accompanying anions. Macrominerals such as sodium and potassium are electrolytes and the body uses electrolytes to maintain acid-base balance and fluid balance (homeostasis) and for normal neurological, myocardial, nerve, and muscle function. Neurons and muscles are activated by electrolyte activity occurring between the extracellular (or interstitial fluid) and intracellular fluid. The macro elements such as calcium and magnesium have been associated with impaired insulin release, insulin resistance, and glucose intolerance in experimental animals and humans. In this review we selected 4 macro elements, namely, sodium, potassium, calcium, and magnesium, because those are the most common and well-studied and correlated with T2D.

### 2.1. Sodium and Potassium

Electrolytes play an important role in many body processes, such as controlling fluid levels, acid-base balance (pH), nerve conduction, and blood clotting and muscle contraction. Electrolyte imbalance resulting from kidney failure, dehydration, and fever and vomiting has been suggested as one of the contributing factors toward complications observed in diabetes and other endocrine disorders [9]. Sodium, which is a predominant extracellular cation, normally in controlled physiological conditions lies in the range of 136–145 mEq/L (136–145 mmol/L) despite large variations in salt and water intake. The normal range of serum potassium value in the serum is very narrow and it ranges from 3.5 to 5 mmol/L [10]. Na^+^/K^+^-ATPase pump is a ubiquitous enzyme that ensures that the transmembrane gradients of sodium and potassium concentrations are maintained. Alterations of this transport system are thought to be linked to several complications of DM, hypertension, and nephropathy [11]. Hyperglycemia sets the internal environment for osmotic diuresis while causing a dilutional effect on electrolyte concentrations. The osmotic effect of glucose results in decreased circulating blood volume and fluid shift from the intracellular spaces causing cellular dehydration. Insulin has been shown to decrease extracellular potassium concentration as well likely through activation of Na^+^/K^+^-ATPase. The synergistic action of cationic imbalance and osmotic effect of glucose could very well impact the course of DM [12, 13]. However, deficiency of insulin or resistance to insulin action may contribute to the development of electrolyte abnormalities. Considering all these factors, the altered distribution of sodium and potassium between the intracellular and extracellular compartments may affect the course of DM as well as its management [14]. Studies on electrolyte imbalances in association with diabetes have reported an inverse relationship between sodium and potassium levels in diabetic coma [15]. This association may be based on the movement of electrolytes between intra- and extracellular space dependent on impaired insulin action [16]. A significant reduction in serum sodium level was reported in T2D patients especially among insulin-treated patients. No significant association was found between T2D and serum potassium [17].

### 2.2. Calcium

Calcium and cyclic AMP are important in the stimulation of insulin release. The increase in the concentration of ionized cytosolic Ca ions directly mediates the effect of glucose to stimulate insulin release from rat islet of Langerhans. Any alterations in calcium flux can have adverse effects on *β*-cell secretory function. Thus it is suggested that inadequate calcium intake could affect the balance between the extracellular and intracellular *β*-cell calcium pools. The normal range of calcium in the serum is from 9 to 10.5 mg/dL (2.2–2.6 mmol/L) [10]. In addition calcium is essential for insulin-mediated intracellular processes in insulin-responsive tissues such as skeletal muscle and adipose tissue; any alteration in calcium may contribute to peripheral insulin resistance via impaired insulin signal transduction, leading to decreased glucose transporter 4 (GLUT4) activity [26]. Role of elevated cytosolic calcium in the pathogenesis of complications in T2D was reported. This is associated with derangements in the regulation of intracellular calcium. Hyperglycemia causes an acute rise in cytosolic calcium due to increased calcium influx and mobilization of intracellular calcium from calcium stores. The stimulation of these calcium channels is mediated by the activation of G protein(s), leading to stimulation of various cellular pathways. Chronic hyperglycemia is also associated with decreased calcium exit from cells. The combination of increased calcium influx and decreased calcium efflux leads to sustained elevation in basal levels of cytosolic calcium [42]. Calcium concentrations in T2D patients with and without complication were significantly lower than those in healthy controls [18].

### 2.3. Magnesium

Magnesium is an essential ion involved in multiple levels in insulin's secretion and its binding and its activity; and it is also a critical cofactor of many enzymes in carbohydrate metabolism [19]. The normal level of serum magnesium ranges between 1.8 and 3.0 mg/dL (0.8–1.2 mmol/L) [10]. Magnesium plays an important role to improve insulin resistance [27]. A randomized controlled trial indicated that oral magnesium supplementation may improve insulin sensitivity even in nondiabetic subjects with normal magnesium status. This emphasizes the need for an early optimization of magnesium intake to prevent insulin resistance and subsequently T2D [43]. The increased incidence of hypomagnesemia among patients with T2D presumably is multifactorial. Altered insulin metabolism, poor glycemic control, and osmotic diuresis may be contributory factors [44]. Hypomagnesemia in diabetes is usually observed in patients with deficient metabolic control, or is associated with DM chronic complications, according to clinical and epidemiological studies [19, 45]. The responsible mechanisms for magnesium deficiency in patients with diabetes have still not been clarified, mainly about the impact in the insulin resistance and in the development of diabetes and its chronic complications [46, 47].

## 3. Trace Elements

The trace element is a dietary mineral that is needed for the proper growth, development, and physiology of the organism. Alterations in the status of trace elements could stem from chronic uncontrolled hyperglycemia. Trace elements include the transition metals vanadium (V), chromium (Cr), manganese (Mn), iron (Fe), cobalt (Co), copper (Cu), zinc (Zn), and molybdenum (Mo) and the nonmetals selenium (Se), fluorine (F), and iodine (I). All of these belong to the category of micronutrients, which are needed by the human body in very small quantities (generally less than 100 mg/day) [48].

### 3.1. Chromium

Chromium is required for normal carbohydrate metabolism and as a critical cofactor for insulin action and is a component of the glucose tolerance factor (GTF), which plays a role in glucose homeostasis [28]. The safe and adequate daily intake of chromium was considered to be in the range 50–200 mg [49]. Normal concentration of chromium in the serum of adult is 0.05–0.5 *μ*g/L (1–10 *μ*mole/L) [50]. Chromium concentrations were significantly reduced in blood of T2D patients as compared to control subjects of both genders but urinary levels of these elements were found to be higher in the diabetic patients than in the age-matched healthy controls [20].

### 3.2. Cobalt

Hyperglycemia is associated with excessive free radical generation and oxidant stress and reduction in the antioxidant status. Normal serum values of cobalt are less than 0.5 *μ*g/L [51]. In an animal study, glycemia-lowering effect of cobalt chloride (CoCl_2_) in diabetic rats decreased the systemic glucose production, increased tissue glucose uptake, or made a combination of the two mechanisms. The action of cobalt resulted in increased expression of glucose transporter 1 (GLUT1) and inhibition of gluconeogenesis in diabetic rats [29]. It is also reported that cobalt alone or with a combination of ascorbate decreases lipid peroxidation in diabetic rats in various organs such as the liver, kidney, heart, and aorta [52]. Compared with nondiabetic subjects serum concentration of cobalt is decreased in T2D [21].

### 3.3. Copper

Copper is considered as both a powerful enzyme catalyst and a dangerous reactant that generates hydroxyl radical. The normal level of total copper in the body ranges between 70 and 140 *μ*g/dL (11–22 *μ*mol/L) [10]. A deficiency of copper results in glucose intolerance, decreased insulin response, and increased glucose response. It is associated with hypercholesterolemia and atherosclerosis. Copper possesses an insulin-like activity and promotes lipogenesis. Recent studies show that no statistical difference was found in the level of copper in both diabetic and healthy patients [20, 22].

### 3.4. Iodine

Iodine is absolutely vital for proper thyroid function. The iodine deficiency is the most common cause of hypothyroidism worldwide. The iodine deficiency will cause mental retardation and cretinism and it is the most devastating in all trace elements [53]. Thyroid hormone controls insulin secretion. In hypothyroidism, there is a reduction in glucose-induced insulin secretion by *β* cells, and the response of *β* cells to glucose or catecholamine is increased in hyperthyroidism due to increased *β*-cell mass. Moreover, insulin clearance is increased in thyrotoxicosis [54]. Insulin resistance and *β*-cell function are inversely correlated with thyroid stimulating hormone (TSH) which may be explained by insulin-antagonistic effects of thyroid hormones along with an increase in TSH. The higher serum TSH usually corresponds to lower thyroid hormones via negative feedback mechanism. As TSH increased, thyroid hormones decreased and insulin antagonistic effects are weakened. These observations demonstrate that insulin imbalance is closely associated with thyroid dysfunction and the phenomenon is mediated via *β*-cell dysfunction [30]. The significantly lower level of iodine was reported in the urine of T2D than in that of healthy control subjects [23].

### 3.5. Iron

Iron is both an essential nutrient and a potential toxicant to cells. The sufficient supply of iron is essential for the functioning of many biochemical processes, including electron transfer reactions, gene regulation, binding and transport of oxygen, regulation of cell growth, and differentiation, and is also involved in the proper function of immune system [55]. The normal range of iron in the adult is 60–170 *μ*g/dL [56]. Impaired glucose metabolism and DM are common clinical manifestations of iron overload in patients with hemochromatosis. Recently, moderately elevated iron stores below the levels commonly associated with hemochromatosis have also been implicated in the etiology of diabetes. Ferritin is a ubiquitous intracellular protein which serves as a marker for tissue iron stores. Levels of plasma ferritin are elevated in persons with prevalent diabetes as compared with nondiabetic controls [57]. Ferritin is also correlated with the prevalence of the metabolic syndrome. In some other studies, ferritin is correlated with the measures of insulin resistance, such as elevated glucose and insulin levels [58, 59]. In addition, two prospective studies have identified an independent association between baseline elevations in iron stores and the incidence of diabetes [60, 61]. Elevated iron stores may induce diabetes through a variety of mechanisms, including oxidative damage to pancreatic *β* cells, impairment of hepatic insulin extraction by the liver, and interference with insulin's ability to suppress hepatic glucose production [31, 32]. In a case-control study it was reported that the association observed between serum ferritin and diabetes risk disappeared after adjustment for components of the metabolic syndrome [62]. But elevated body iron stores below the levels observed in haemochromatosis are associated with higher risk of T2D independently of established risk factors and a range of diabetes biomarkers. However, soluble transferrin receptor levels were not associated with T2D and high ferritin levels are related to a higher risk of T2D [24].

### 3.6. Selenium

Selenium, a trace element, is involved in the complex system of defense against oxidative stress through selenium-dependent glutathione peroxidases and other selenoproteins [63]. The normal selenium concentration in the serum is less than 8 *μ*g/dL [64]. Due to its antioxidant properties, selenium might be preventing the development of diabetes. In addition, selenate, an inorganic form of selenium, mimics insulin activity in experimental models [33]. Selenium is known to act as an antioxidant and peroxynitrite scavenger when incorporated into selenoproteins. This antioxidant property of selenium prevents the development of complications in diabetic patients [65]. While in other studies higher serum selenium concentrations were associated with a higher prevalence of diabetes [34], in a recent study the mean selenium concentrations in T2D patients with and without complication were significantly lower than those in healthy controls [18].

### 3.7. Manganese

Manganese plays an important role in a number of physiologic processes as a constituent of some enzymes such as pyruvate carboxylase and arginase and an activator of different enzymes such as phosphoenolpyruvate carboxykinase (PEPCK) and glutamine synthetase. These manganese activated enzymes play important roles in the metabolism of carbohydrates, aminoacids, and cholesterol. Manganese helps in glucose metabolism and it is required for normal synthesis and secretion of insulin [35]. The normal range of manganese in the adult blood is from 0.59 to 0.75 *μ*g/L [50]. The level of manganese is lower in T2D subjects as compared to control subjects [66]. In an another study, the mean manganese was significantly low in blood and scalp-hair samples of diabetic patients as compared to control and both genders [20].

### 3.8. Zinc

Zinc plays an important role in glucose metabolism [36]. It helps in the utilization of glucose by muscle and fat cells. It is required as a cofactor for the function of intracellular enzymes that may be involved in protein, lipid, and glucose metabolism. Zinc may be involved in the regulation of insulin receptor-initiated signal transudation mechanism and insulin receptor synthesis [37]. Zinc is a structural part of key antioxidant enzymes such as superoxide dismutase, and zinc deficiency impairs their synthesis, leading to increased oxidative stress [67]. Zinc has a biphasic effect in that it is required for insulin storage and cellular binding, although high concentrations can lead to a reduction in insulin release [68].

The normal range of zinc in serum/plasma is reported as 84–159 *μ*g/dL [68]. The antigenic properties of zinc affect insulin binding to hepatocyte membranes and a deficiency may lead to increased insulin resistance and hyperglycemia. Elevated glucose in turn produces hyperzincuria. Low zinc has also been seen to lead to poor or slowed wound healing, which is common in diabetic patients [68]. Oxidative stress plays an important role in the pathogenesis of diabetes and its complications. Clinical studies reported that serum levels of zinc are usually found low in T2D patients compared to nondiabetic due to the impaired intestinal reabsorption of endogenous zinc and the increase in excretion of zinc into the intestine during the digestive process may lead to this low serum zinc level [25]. The zinc supplementation in patients with T2D improved insulin secretion, while suppressing glucagon and glucose-6-phosphatase levels [69]. In certain studies, the effect on serum insulin by zinc supplementation has been contradicted [70].

### 3.9. Vanadium

Vanadium affects various aspects of carbohydrate metabolism including glucose transport, glycolysis, glucose oxidation, and glycogen synthesis [38, 39]. Vanadium exists in several valence states, with vanadate (+4) and vanadyl (+5) forms being the most common in biological systems. In animal models, vanadium has been shown to facilitate glucose uptake and metabolism, facilitate lipid and amino acid metabolism, improve thyroid function, enhance insulin sensitivity, and negatively affect bone and tooth development in high doses. Vanadium acts primarily as an insulin mimetic agent, although enhanced insulin activity and increased insulin sensitivity have also been noted. More recent research suggests that insulin may be required for its effects [40, 41].

Vanadium appears to affect several points in the insulin signaling pathway and may lead to upregulation of the insulin receptor and subsequent intracellular signaling pathways. Suggested effects include insulin receptor autophosphorylation, increased protein tyrosine and serine threonine kinase activity, inhibition of phosphotyrosine phosphatase activity, increased adenylate cyclase activity, altered glucose-6-phosphatase activity, inhibition of hepatic gluconeogenesis, and increased glycogen synthesis [40, 41].

The normal range of vanadium in blood or serum is from 17 to 118 ng/L [71]. An elevated vanadium level is also reported in diabetic persons in a study with different blood fractions [22]. In subjects with T2D, vanadium increased insulin sensitivity, glucose oxidation and glycogen synthesis were increased, and hepatic glucose output was suppressed [72, 73]. One of the obstacles in using vanadium for glucose management is that it is known to be harmful to humans. Glutamate pyruvate transaminase is an enzyme used to monitor liver function and if the levels of this enzyme in the plasma are raised, it indicates liver cell damage [74].

## 4. Conclusion

Micro-/macronutrients play an important role in glucose metabolism, so understanding the impact of micronutrient deficiencies and the potential utility of supplementation is relevant to the prevention and/or management of type 2 diabetes mellitus. Macro elements are the natural elements of which the body needs more amounts and are more important than any other minerals. Trace elements are required in minute amounts to maintain a healthy body. They are required mainly as components of enzymes and hormones or are involved in the activation of enzymes. Electrolyte imbalance in diabetes is primarily a result of elevated blood glucose. With hyperglycemia, the body tries to rid itself of the excess blood glucose by increasing urinary output. Increased urination produces water and electrolyte loss, which then upsets the body's balance of electrolytes. The balance is especially disturbed between sodium and potassium. Hypomagnesemia in diabetes is usually observed in patients with deficient metabolic control or is associated with the DM chronic complications, according to clinical and epidemiological studies. The responsible mechanisms for Mg deficiency in patients with diabetes have still not been clarified, mainly about the impact in the insulin resistance and in the development of diabetes and its chronic complications. Any alterations in calcium flux can have adverse effects on *β*-cell secretory function. The elevated cytosolic calcium will lead to the pathogenesis of complications of T2D. Chromium is required for normal carbohydrate metabolism and plays a role in glucose homeostasis. The effect of cobalt in diabetes causes decreases in systemic glucose production and increased tissue glucose uptake. A deficiency of copper results in glucose intolerance, decreased insulin response, and increased glucose response. Copper possesses an insulin-like activity and promotes lipogenesis. The role of iodine is correlated with thyroid hormone and it is clear that insulin resistance and *β*-cell function are inversely correlated with thyroid stimulating hormone which may be explained by insulin-antagonistic effects of thyroid hormones along with an increase in thyroid stimulating hormone (TSH). Elevated iron stores may induce diabetes through a variety of mechanisms, including oxidative damage to pancreatic *β* cells, impairment of hepatic insulin extraction by the liver, and interference with insulin's ability to suppress hepatic glucose production. The effect of selenium in diabetes has contradictory effects; the antioxidant property of selenium prevents the development of complications in diabetic patients. While in other studies higher serum selenium concentrations were associated with a higher prevalence of diabetes. Manganese activated enzymes plays an important role in the metabolism of carbohydrates, aminoacids, and cholesterol and it is required for normal synthesis and secretion of insulin. Many of the complications of diabetes may relate to an increase in intracellular oxidant and free radicals associated with decrease in intracellular zinc and zinc dependent antioxidant enzymes. The vanadium salt was shown to induce a mechanism to reduce hyperglycemia and improve insulin action by increasing the glucose transporters activity via insulin receptor substrates 1 and 2 (IRS1/2) and phosphatidylinositol 3-kinase (PI 3-kinase). Nutrition management aims to improve health quality maintaining blood glucose levels in normal range so as to reduce the risk for diabetes complications. A well-balanced diet will maintain the impairment of essential macro- and micronutrients in patient with diabetes. In this paper, micronutrients recommendations have been displayed for the management of T2D and the prevention of its complications.

## Figures and Tables

**Table 1:** Micronutrient status in Type 2 diabetes subjects.

Micronutrients	Status in T2D subjects	Reference
Calcium	Low	[18]
Magnesium	Low	[19]
Sodium	Low	[17]
Potassium	No effect	[17]
Chromium	Low	[20]
Cobalt	Low	[21]
Copper	No effect	[20, 22]
Iodine	Low	[23]
Iron	High	[24]
Selenium	Low	[18]
Manganese	Low	[20]
Zinc	Low	[25]
Vanadium	High	[22]

**Table 2:** Micronutrient functions in type 2 diabetes.

Trace/macro elements	Functions in type 2 diabetes
Sodium and potassium	Na^+^/K^+^− ATPase pump is a ubiquitous enzyme that ensures that the transmembrane gradients of sodium and potassium concentrations are maintained. Alterations of this transport system are thought to be linked to several complications of diabetes mellitus [11].

Calcium	Any alterations in calcium flux can have adverse effects on *β*-cell secretory function and may interfere with normal insulin release, especially in response to a glucose load. The elevated cytosolic calcium will lead to the pathogenesis of complications of T2D which in turn may interfere with normal insulin release, especially in response to a glucose load [26].

Magnesium	The magnesium is an essential ion involved in multiple levels in insulin's secretion and its binding and its activity; and it is also a critical cofactor of many enzymes in carbohydrate metabolism. The magnesium plays an important role to improve insulin resistance [19, 27].

Chromium	The chromium is required for normal carbohydrate metabolism and as a critical cofactor for insulin action and is a component of the glucose tolerance factor (GTF), which plays a role in glucose homeostasis [28].

Cobalt	The glycemia-lowering effect of cobalt chloride (CoCl_2_) decreased systemic glucose production, increased tissue glucose uptake, or made a combination of the two mechanisms. The action of cobalt results increased expression of glucose transporter 1 (GLUT1) and inhibition of gluconeogenesis [29].

Copper	A deficiency of copper results in glucose intolerance, decreased insulin response, and increased glucose response. It is associated with hypercholesterolemia and atherosclerosis. The copper possesses an insulin-like activity and promotes lipogenesis [20, 22].

Iodine	The role of iodine is correlated with thyroid hormone and it is clear that insulin resistance and *β*-cell function are inversely correlated with thyroid stimulating hormone which may be explained by insulin-antagonistic effects of thyroid hormones along with an increase in thyroid stimulating hormone (TSH) [30].

Iron	Elevated iron stores may induce diabetes through a variety of mechanisms, including oxidative damage to pancreatic *β* cells, impairment of hepatic insulin extraction by the liver, and interference with insulin's ability to suppress hepatic glucose production [31, 32].

Selenium	The effect of selenium in diabetes has contradictory effects; the antioxidant property of selenium prevents the development of complications in diabetic patients. While in other studies higher serum selenium concentrations were associated with a higher prevalence of diabetes [33, 34].

Manganese	The enzyme which is activated by manganese plays important roles in the metabolism of carbohydrates, amino acids, and cholesterol and it is required for normal synthesis and secretion of insulin [35].

Zinc	The zinc plays an important role in glucose metabolism. It helps in the utilization of glucose by muscle and fat cells. It is required as a cofactor for the function of intracellular enzymes that may be involved in protein, lipid, and glucose metabolism. The zinc may be involved in the regulation of insulin receptor-initiated signal transudation mechanism and insulin receptor synthesis [36, 37].

Vanadium	The vanadium affects various aspects of carbohydrate metabolism including glucose transport, glycolysis, and glucose oxidation and glycogen synthesis. The vanadium acts primarily as an insulin mimetic agent, although enhanced insulin activity and increased insulin sensitivity have also been noted [38–41].

## Abbreviations

IDF: International Diabetes Federation
T1D: Type 1 diabetes
T2D: Type 2 diabetes
GDM: Gestational diabetes mellitus
DM: Diabetes mellitus
GLUT 4: Glucose transporter 4
GTF: Glucose tolerance factor
GTUT1: Glucose transporter 1
TSH: Thyroid stimulating hormone
PEPCK: Phosphoenolpyruvate carboxykinase
IRS1/2: Insulin receptor substrates 1 and 2
PI 3-kinase: Phosphatidylinositol 3-kinase.
